# The *Escherichia coli* GcvB sRNA Uses Genetic Redundancy to Control *cycA* Expression

**DOI:** 10.5402/2012/636273

**Published:** 2012-05-28

**Authors:** Lorraine T. Stauffer, George V. Stauffer

**Affiliations:** Department of Microbiology, University of Iowa, Iowa City, IA 52242, USA

## Abstract

The *Escherichia coli* sRNA GcvB regulates several genes involved in transport of amino acids and peptides (*sstT, oppA, dppA*, and *cycA*). Two regions of GcvB from nt +124 to +161 and from nt +73 to +82 are complementary with essentially the same region of the *cycA* mRNA. Transcriptional fusions of *cycA* to *lacZ* showed the region of *cycA* mRNA that can pair with either region of GcvB is necessary for regulation by GcvB. However, mutations in either region of *gcvB* predicted to disrupt pairing between *cycA* mRNA and GcvB did not alter expression of a *cycA-lacZ* translational fusion. A genetic analysis identified nts in GcvB necessary for regulation of the *cycA-lacZ* fusion. The results show that either region of GcvB complementary to *cycA* mRNA can basepair with and independently repress *cycA-lacZ* and both regions need to be changed to cause a significant loss of repression.

## 1. Introduction

 The *E*. *coli gcvB* gene encodes a sRNA of 206 nts [1]. Transcription of *gcvB* is activated by GcvA when cellular glycine is high and repressed by GcvA when glycine is limiting; repression by GcvA requires the accessory GcvR protein [1]. GcvB regulates *cycA*, encoding the glycine transport protein [2]. Thus, GcvB regulates its own synthesis by controlling the level of glycine transported into the cell. A Δ*gcvB* strain shows constitutive synthesis of OppA and DppA, the periplasmic binding protein components of the two major peptide transport systems, SstT, a serine transport system, and CycA, a glycine transport system [1–4]. The *Salmonella enterica *serovar Typhimurium GcvB also regulates OppA and DppA levels and several other genes involved in transport of polar and branched amino acids and general amino acid metabolism [5, 6].

 Evidence suggests GcvB regulates its target mRNAs by an antisense mechanism, basepairing with the mRNAs to prevent translation initiation [3–6]. Although it is unclear how extensive pairing between a sRNA and a mRNA must be, research indicates one or two regions of 8-9 basepairs is sufficient for regulation [7]. In cases where basepairing interactions occur, the RNA chaperone Hfq is required, likely to alter RNA secondary structures or to bring together sRNAs and target mRNAs, increasing local RNA concentrations [8–11]. Hfq binds GcvB [11, 12], stabilizing the RNA [5, 13], and loss of Hfq results in the loss of repression of GcvB target mRNAs [2, 4, 5, 13]. For sRNAs studied in detail that regulate by an antisense mechanism, often a single basepair change in the sRNA or its target mRNA results in a loss of regulation by the sRNA (e.g., the sRNA SgrS and its target *ptsG* mRNA [14]). For GcvB, however, it is surprising that most changes predicted to disrupt pairing with regions of the target mRNAs have little or no effect on GcvB's ability to regulate [2–4].

**Figure 1:**
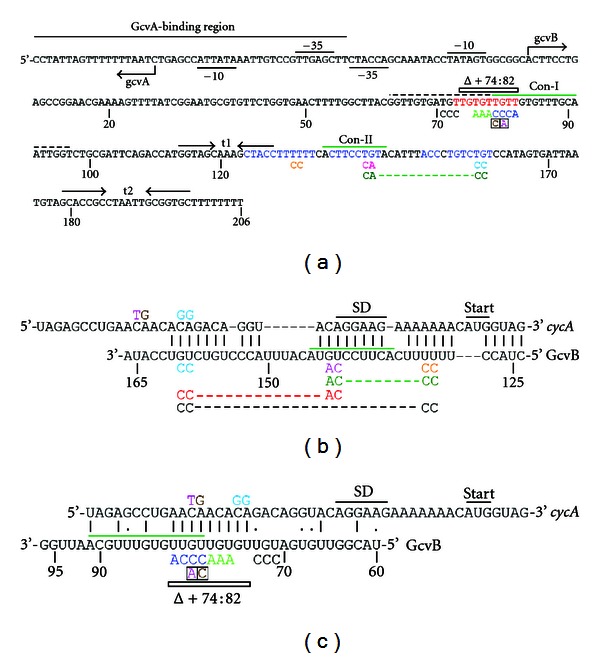
(a) The *gcvA*/*gcvB* promoter region and *gcvB* gene. Promoter −35 and −10 elements are underlined for *gcvA* and overlined for *gcvB* [1, 15]. The GcvA-binding site is indicated by a line [16]. Inverted arrows show stem-loop sequences of putative transcription terminator t1 and terminator t2. A 13 base and a 10 base conserved sequence in *gcvB* homologs are designated Con-I and Con-II (green bars) [1, 3, 5]. Con-I is part of a larger G/T-rich domain (dashed line) essential for interaction of GcvB with most characterized target mRNAs [4, 5, 13]. Bases in GcvB complementary with *cycA* mRNA in the +73 to +82 region are in red and in the +124 to +161 region in blue. Changes in *gcvB* shown not to alter *cycA*-*lacZ* expression are below the sequence and are color coded [2]. Two independent changes isolated using p*gcvB^+142CA+159CC^* as template and that result in loss of GcvB repression of *cycA*-*lacZ* are boxed. (b) Comparison of GcvB from nt +124 to +166 with *cycA* mRNA. (c) Comparison of GcvB from nt +60 to +96 with *cycA* mRNA. For (b) and (c), complementarity is indicated with lines and GU bps with dots. Changes in *gcvB* are shown below the sequences, and changes in *cycA* are shown above the sequences and are color coded (see text for details).

 GcvB homologs contain two conserved sequences of 13 nts (Con-I) and 10 nts (Con-II) (Figure 1(a)) [1, 3, 5]. In addition, a G/T-rich domain that includes the Con-I sequence was shown to be essential for interaction with most GcvB target mRNAs in *E*. *coli* and *S*. *enterica* [4, 5, 13]. In *S*. *enterica*, the Con-II region also pairs with *cycA* mRNA, possibly inhibiting translation initiation [6]. Analysis of *E*. *coli* GcvB identified two regions from nt +73 to +82 and from nt +124 to +161 complementary to *cycA* mRNA (Figures 1(b) and 1(c)). The region from +73 to +82 overlaps Con-I and the G/T-rich domain, and the region from +124 to +161 overlaps Con-II (Figure 1(a)). In addition, transcriptional fusions of *cycA* to *lacZ* verified the region from −8 to −26 upstream of the AUG start codon, and complementary with both the +73 to +82 and +124 to +161 regions of GcvB is required for regulation of *cycA* (Figures 1(b) and 1(c)) [2]. However, changes in either region of GcvB independently did not alter regulation of *cycA*-*lacZ* [2]. We devised a genetic selection to identify any nts in GcvB required to regulate *cycA*-*lacZ*. In this study, we show the region of GcvB from +73 to +82 as well from nt +124 to +161 is important for regulation of a *cycA*-*lacZ* fusion. In addition, both regions can independently repress, suggesting GcvB regulates *cycA*-*lacZ* by a mechanism that uses redundancy within GcvB.

## 2. Materials and Methods

### 2.1. Bacterial Strains, Plasmids, and Phage

 The *E*. *coli* strains, plasmids, and phage used are listed in Table 1 or are described in the text.

### 2.2. Media

 The complex medium used was Luria-Bertani broth (LB) [19]. Agar was added at 1.5% (w/v) to make solid medium. The defined medium used was the salts of Vogel and Bonner [20] supplemented with 0.4% (w/v) glucose (GM). Ampicillin (Amp) was added at 50 *μ*g mL^−1^. X-gal was added at 40 *μ*g mL^−1^.

### 2.3. DNA Manipulation

Plasmid DNA was isolated using a QIAprep Spin Miniprep Kit (Qiagen, Santa Clara, CA). Vent DNA polymerase, Taq DNA polymerase, and restriction enzymes were from New England Biolabs, Inc. (Beverly, MA). T4 DNA ligase was from Roche Diagnostics (Indianapolis, IN). Reactions were as described by the manufacturers.

### 2.4. Enzyme Assay

*β*-Galactosidase assays were performed on mid-log phase cells (OD_600_~0.5) using the chloroform/SDS lysis procedure [19]. Results are the averages of two or more assays with each sample done in triplicate. Results were analyzed using the Student's *t*-test.

### 2.5. Random and Site-Directed Mutagenesis of *gcvB*

 Plasmid pGS634 carries the *gcvB* ^+142CA+159CC^ allele on an *Eco*RI-*Hind*III fragment [2]. Using pGS634 as template, error-prone PCR was used [21] to amplify DNA containing *gcvB*. The upstream primer (GcvB-For) was 5′-CTAGGCGGAATTCGCGGTGGTAATCGTTTAGACATGGC with an *Eco*RI site (underlined) and hybridizes 50 bps upstream of the *gcvB* transcription start site. The downstream primer (GcvB-Rev) was 5′-GGGGAAGCTTGAAAGAGATGGTCGAACTGG with a *Hin*dIII site (underlined) and hybridizes to pGS634 beginning 44 bps after the *gcvB* transcription stop site. The 423 bp amplified DNA fragment was digested with *Eco*RI + *Hin*dIII, cloned into *Eco*RI-*Hin*dIII digested and gel-purified vector pGS341 [17], replacing the WT *gcvA* gene, and transformed into the Δ*gcvB* strain GS1144 lysogenized with *λcycA*-*lacZ*. After 1 round of Amp counterselection [19], cells were plated on LB plates + Amp + X-gal. Killing nontransformed lysogens made identification of darker blue colonies efficient. Plasmid DNA was prepared from potential mutants (dark blue transformants) and the DNA sequenced at the Core Facility at the University of Iowa to verify mutations.

 Site-directed mutagenesis of *gcvB* was performed using the PCR “megaprimer” procedure [22] with pGS594 (p*gcvB* ^+^) as template. Changes were verified by DNA sequence analysis and are predicted by the mfold program [23, 24] to leave the GcvB secondary structure intact.

### 2.6. Construction of the *gcvB* ^t1↑^ Allele and *gcvB* ^t1↑^ Allele + Additional Mutations

**Figure 2:**
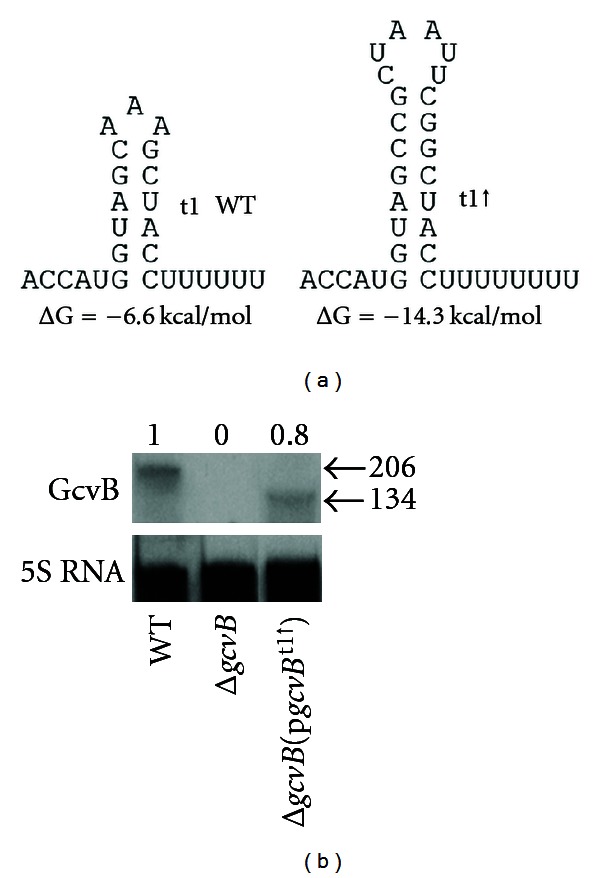
(a) WT terminator t1 and changes in t1 predicted to increase (↑) transcription termination. Primers used to construct the t1↑ allele delete the sequence distal to the t1↑ changes. (b) Northern analysis of GcvB. RNA was isolated from WT, Δ*gcvB,* or Δ*gcvB* transformed with a single-copy plasmid carrying the *gcvB* ^t1↑^ allele and probed with either a DIG-labeled GcvB or 5S rRNA-specific DNA probe. Numbers above each lane indicate levels of GcvB relative to WT.

The *gcvB* ^t1↑^ allele with bp changes that make a strong transcription terminator at t1 and removes sequence distal to t1 was constructed using p*gcvB* ^+^ as template and upstream primer GcvB-For and downstream primer GcvB-t1↑ 5′-GGGGAAGCTTGAAAAAAAAGGTAGCCGAATTAGCGGCTACCATGGTCTGAATCGCAG with a *Hin*dIII site (underlined) and that hybridizes beginning at bp +135 in *gcvB*. The amplified DNA was digested with *Eco*RI + *Hin*dIII, cloned into *Eco*RI-*Hin*dIII digested and gel-purified vector pGS341, replacing the WT *gcvA* gene. Base changes were verified by DNA sequence analysis and the plasmid-designated pGS642 (p*gcvB* ^t1↑^) (Figure 2(a)). Mutations in *gcvB* were then combined with the *gcvB* ^t1↑^ allele by PCR. Plasmids pGS596 (p*gcvB* ^+71CCC^), pGS602 (p*gcvB* ^+76AAA^), pGS629 (p*gcvB* ^+79CCCA^), pGS644 (p*gcvB* ^+142CA+159CC+79C^), and pGS645 (p*gcvB* ^+142CA+159CC+80A^) were used as templates with upstream primer GcvB-For and downstream primer GcvB-t1↑. The amplified DNA fragments were cloned as described for the p*gcvB* ^t1↑^ allele. Changes were verified by DNA sequence analysis. The plasmids were designated pGS647 (p*gcvB* ^t1↑+71CCC^), pGS649 (p*gcvB* ^t1↑+76AAA^), pGS653 (p*gcvB* ^t1↑+79CCCA^), pGS655 (p*gcvB* ^t1↑+79C^), and pGS656 (p*gcvB* ^t1↑+80A^), respectively (Figure 1).

### 2.7. Construction of the *gcvB* ^Δ+74 : 82^ Allele and *gcvB* ^Δ+74 : 82^ Allele + Additional Mutations

The *gcvB* ^Δ+74 : 82^ allele with a deletion from bp +74 to +82 was constructed using the PCR “megaprimer” procedure [22]. The new plasmid was designated pGS680 (p*gcvB* ^Δ+74 : 82^) (Figures 1(a) and 1(c)). Base changes were verified by DNA sequence analysis. Mutations in *gcvB* in the +124 to +161 region were then combined with the *gcvB* ^Δ+74 : 82^ allele by the PCR “megaprimer” procedure [22]. The new plasmids were designated pGS682 (p*gcvB* ^Δ+74 : 82+142CA^), pGS683 (p*gcvB* ^Δ+74 : 82+159CC^), pGS684 (p*gcvB* ^Δ+74 : 82+131CC^), pGS697 (p*gcvB* ^Δ+74 : 82+142CA+159CC^), pGS698 (p*gcvB* ^Δ+74 : 82+131CC+142CA^), and pGS699 (p*gcvB* ^Δ+74 : 82+131CC+159CC^) (Figure 1).

### 2.8. Construction of *λcycA* ^−24GG^-*lacZ*, *λcycA* ^−29G^-*lacZ*, and *λcycA* ^−30T^-*lacZ* Mutations

 Plasmid p*cycA*-*lacZ* carries an *E*. *coli cycA*-*lacZ *translational fusion [2]. Using p*cycA*-*lacZ* as template, PCR “megaprimer” mutagenesis [22] was used to create changes in *cycA*-*lacZ* (Figures 1(b) and 1(c)). Base changes were verified by DNA sequence analysis at the DNA Core Facility of the University of Iowa. The intermediate plasmids were designated p*cycA* ^−24GG^-*lacZ*, p*cycA* ^−29G^-*lacZ,* and p*cycA* ^−30T^-*lacZ*. A 5,788 bp *Eco*RI-*Mfe*I fragment from each plasmid carrying the mutant *cycA*-*lacZ* fusions and *lacYA* genes was then ligated into the *Eco*RI site of phage *λ*gt2 [18]. The new phage was designated *λcycA* ^−24GG^-*lacZ*, *λcycA* ^−29G^-*lacZ,* and *λcycA* ^−30T^-*lacZ*. The phage were used to lysogenize appropriate *E*. *coli* host strains as described previously [25]. Each lysogen was tested to ensure that it carried a single copy of the *λ* chromosome by infection with *λc*I90*c*17 [26]. All lysogens were grown at 30°C since all fusion phages carry the *λc*I857 mutation, resulting in a temperature sensitive *λc*I repressor [18].

### 2.9. RNA Isolation and Northern Analysis

*E. coli* strains were grown in 5 mL of LB to mid-log phase. Total RNA was isolated using an RNeasy Mini Kit (Qiagen, Santa Clara, CA) and quantified using a NanoDrop ND-1000 Spectrophotometer. Northern analysis and quantification of RNA were performed as described [13].

## 3. Results

### 3.1. Nucleotides in GcvB Important for *cycA-lacZ* Repression

 It was suggested that in *S*. *enterica* several regions in GcvB can independently block translation initiation of *cycA* mRNA [6]. To identify any sequence in *E*. *coli* GcvB required to regulate *cycA*-*lacZ*, we devised a genetic selection. Since two regions of *GcvB* from nt +73 to +82 and from +124 to +161 are complementary to *cycA* mRNA (Figures 1(b) and 1(c)), we biased the selection by disrupting the primary pairing interactions between GcvB and *cycA* mRNA. If both regions are able to pair with *cycA* mRNA, disrupting the primary region of interaction would increase the chances of identifying additional nts important for repression. Starting with pGS634 (p*gcvB* ^+142CA+159CC^) as template error-prone PCR was used to mutagenize *gcvB* [21]. Transformation of a Δ*gcvB* strain with the mutagenized DNA allowed us to identify two mutants with increased *cycA*-*lacZ* expression (darker blue colonies on X-gal plates). Plasmid DNA prepared from the mutants was sequenced, and two changes in *gcvB* were identified, a -T- to -C- change at nt +79 and a -G- to -A- change at nt +80 (Figure 1(a), boxed nts). The new plasmids were designated pGS644 (p*gcvB* ^+142CA+159CC+79C^) and pGS645 (p*gcvB* ^+142CA+159CC+80A^).

**Figure 3:**
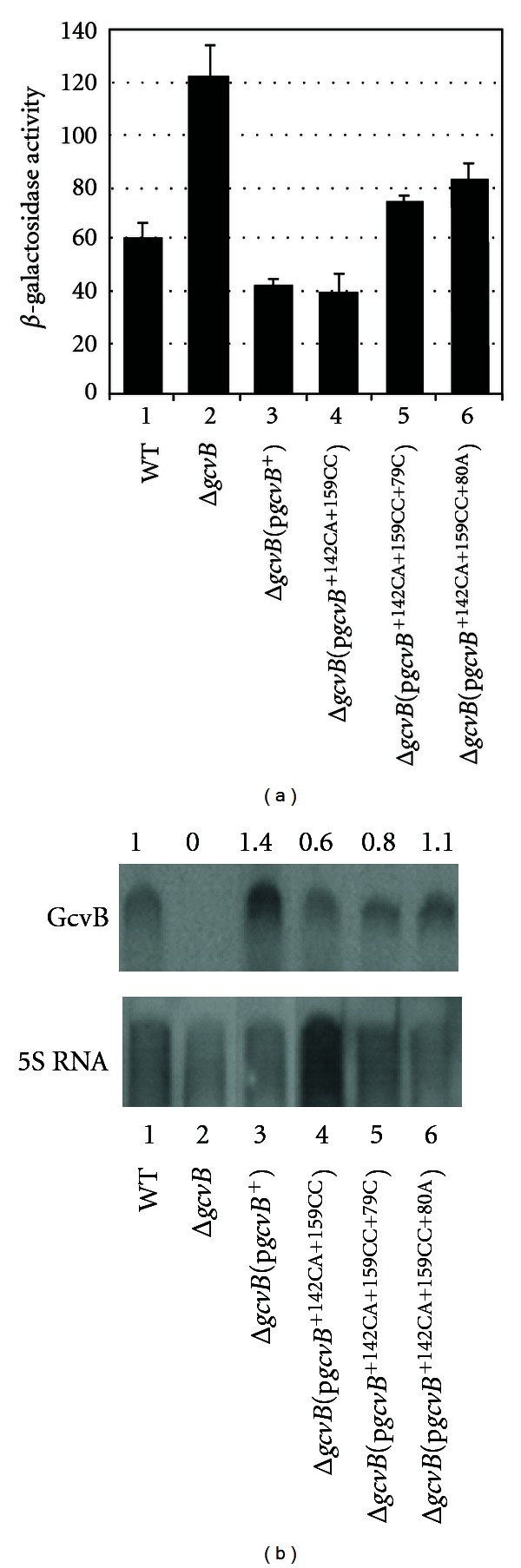
(a) Effects of *gcvB* mutant alleles on *cycA*-*lacZ* expression. WT and Δ*gcvB* *λcycA*-*lacZ* lysogens transformed with the indicated *gcvB* alleles were grown in LB (+Amp for transformants) to mid-log phase and assayed for *β*-galactosidase. (b) Northern analysis of GcvB. RNA was isolated from WT, Δ*gcvB,* or Δ*gcvB* transformed with the indicated *gcvB *alleles and probed with either a DIG-labeled GcvB or 5S rRNA specific DNA probe. Numbers above each lane indicate levels of GcvB relative to WT.

 To determine the effects of the mutations on *cycA*-*lacZ* expression, the Δ*gcvB* *λcycA*-*lacZ* lysogen was transformed with the new plasmids and control plasmid p*gcvB* ^+^ and assayed for *β*-galactosidase. *β*-galactosidase levels were 2-fold higher in the Δ*gcvB* lysogen compared to WT and repression was restored in the Δ*gcvB*[p*gcvB* ^+^] transformant (Figure 3(a), lanes 1, 2, and 3). In addition, as reported [2], the p*gcvB* ^+142CA+159CC^ allele repressed *cycA*-*lacZ* as well as WT *gcvB* ^+^ (Figure 3(a), lane 4). In the presence of the p*gcvB* ^+142CA+159CC+79C^ and p*gcvB* ^+142CA+159CC+80A^ alleles, *β*-galactosidase levels were about 2-fold higher than in the control strains (Figure 3(a), compare lanes 3 and 4 with lanes 5 and 6). Of interest, changes at +79 and +80 (although different nts than the +79C and +80A changes) had no effect on *cycA*-*lacZ* expression in the absence of the *gcvB* ^+142CA+159CC^ mutation [2], suggesting both regions must be altered to see a loss of GcvB repression.

 To determine if each *gcvB* allele produced comparable levels of GcvB, a Northern analysis was performed. The results showed about the same levels of GcvB for each RNA sample tested except the *gcvB* ^+142CA+159CC^ allele, which had about 60% of the WT level (Figure 3(b)). However, the *gcvB* ^+142CA+159CC^ allele showed normal repression of *cycA*-*lacZ* (Figure 3(a), lane 4). Thus, loss of repression for the *gcvB* ^+142CA+159CC+79C^ and *gcvB* ^+142CA+159CC+80A^ alleles is not due to reduced levels of the mutant RNAs.

### 3.2. Sequence Preceding Terminator t1 Is Able to Repress *cycA-lacZ*

**Figure 4:**
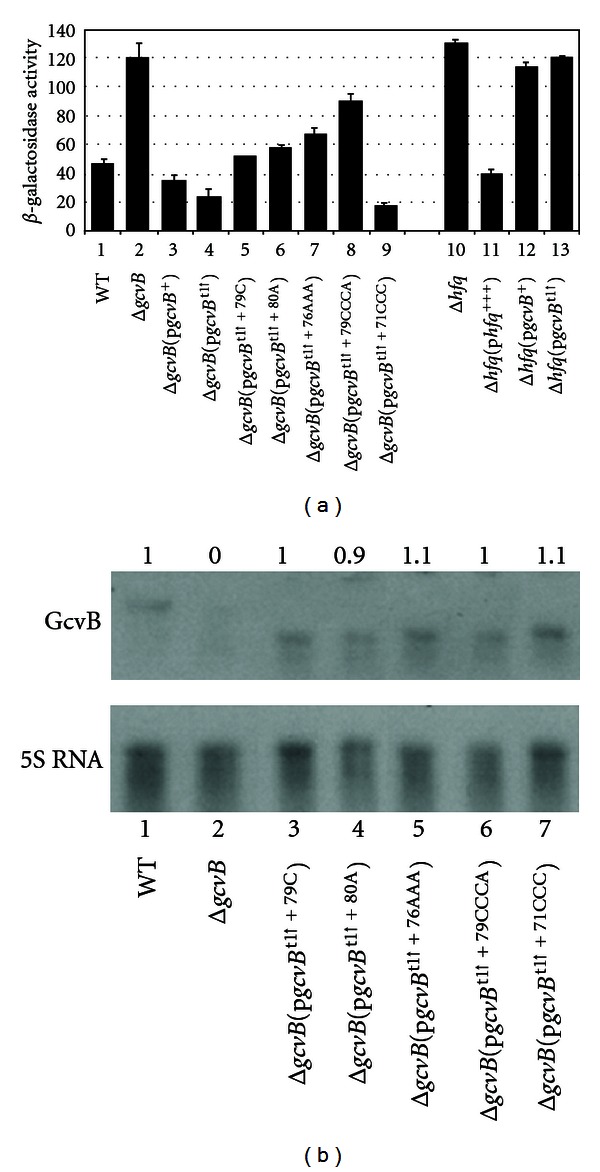
(a) Effects of the *gcvB* ^t1↑^ allele plus additional changes on *cycA*-*lacZ* expression. WT, Δ*gcvB* and Δ*hfq* *λcycA*-*lacZ* lysogens transformed with the indicated plasmids were grown in LB (+Amp for transformants) to mid-log phase and assayed for *β*-galactosidase. (b) Northern analysis of GcvB. RNA was isolated from WT, Δ*gcvB* or Δ*gcvB* transformed with the indicated plasmids and probed with either a DIG-labeled GcvB or 5S rRNA specific DNA probe. Numbers above each lane indicate levels of GcvB relative to WT.

 One possibility that could explain the above results is either region of GcvB complementary to the *cycA* mRNA is sufficient to cause repression and both regions must be changed to see an effect. Two experiments provide results that support this hypothesis. Two Rho-independent terminator sequences can be found in *gcvB* centered at bp +121 and +189/190, designated t1 and t2, respectively (Figure 1(a)) [1]. Although *in vivo* and *in vitro* evidence suggests some termination occurs at t1 [1], no short transcript was detected in either *E*. *coli* or *S*. *enterica* by Northern analysis [5, 13]. We constructed a *gcvB* allele where t1 is a better Rho-independent terminator (*gcvB* ^t1↑^) (Figure 2(a)). If either region of GcvB complementary to *cycA* mRNA can pair with the mRNA to cause repression, elimination of sequence distal to t1 should still result in repression of *cycA*-*lacZ*. To ensure any regulation observed is not due to read-through of the *gcvB* ^t1↑^ allele, all sequence following t1 was deleted (see Materials and Methods). A Northern Blot showed the *gcvB* ^t1↑^ allele produced only a short RNA of ~134 nts and at levels about 80% of the WT level (Figure 2(b)). Thus, any change in regulation of *cycA*-*lacZ* is likely due to the short RNA rather than a change in the synthesis or stability of the RNA. *β*-Galactosidase levels were 2.4-fold higher in the Δ*gcvB* lysogen compared to WT, and repression was restored in the Δ*gcvB*[p*gcvB* ^+^] complemented strain (Figure 4, compare lanes 1, 2, and 3). The *gcvB* ^t1↑^ allele showed ~1.5-fold better repression of *cycA*-*lacZ* than the WT *gcvB* allele (Figure 4, lanes 3 and 4). Although the change was small, it is statistically significant (*P* value = 0.02 relative the p*gcvB* ^+^ transformant). The results suggest the region distal to terminator t1 is not necessary for GcvB repression of *cycA*-*lacZ*. 

 Next, we introduced the +79C and +80A changes, as well as several other changes that do not alter *cycA*-*lacZ* expression in the full length GcvB, into the *gcvB* ^t1↑^ allele. The +79C (brown), +80A (purple), +76AAA (green), and +79CCCA (blue) changes reduce complementarity of GcvB with *cycA* mRNA (Figure 1(c)) and resulted in reduced repression of *cycA*-*lacZ* when combined with the *gcvB* ^t1↑^ allele (Figure 4(a), compare lane 4 with lanes 5–8). The +71CCC change (black) increases complementarity between GcvB and *cycA* mRNA (Figure 1(c)) and resulted in 1.3-fold increased repression (Figure 4(a), compare lanes 4 and 9). Although the change is small, it is statistically significant (*P* value = 0.005 relative to the p*gcvB* ^t1↑^ transformant). A Northern analysis showed about the same amounts of GcvB for each of the RNA samples tested (Figure 4(b)), suggesting altered regulation is not due to altered levels of the mutant RNAs. The results show the region from +70 to +90 is sufficient for GcvB regulation of *cycA*-*lacZ*, but changes in this region only result in altered regulation if the region distal to t1 is changed or deleted.

### 3.3. The *gcvB* ^t1↑^ Allele Is Dependent on Hfq

 It is possible that the truncated GcvB is able to regulate independently of Hfq. To test this possibility, we transformed the Δ*hfq* strain with p*hfq* ^3+^, p*gcvB* ^+^, and p*gcvB* ^t1↑^ alleles and assayed for *β*-galactosidase activity. As shown previously [2], the Δ*hfq* *λcycA*-*lacZ* lysogen showed high levels of *β*-galactosidase activity and repression was restored in the Δ*hfq*[p*hfq* ^3+^] complemented strain (Figure 4(a), lanes 10 and 11). Both the Δ*hfq*[p*gcvB* ^+^] and Δ*hfq*[p*gcvB* ^t1↑^] transformants showed high levels of *β*-galactosidase activity, suggesting the truncated GcvB still requires Hfq for repression of *cycA*-*lacZ* (Figure 4(a), lanes 12 and 13). The results indicate that the Hfq-binding site for GcvB occurs in the region preceding terminator t1.

### 3.4. Sequence Distal to Terminator t1 Is Able to Repress *cycA-lacZ*

**Figure 5:**
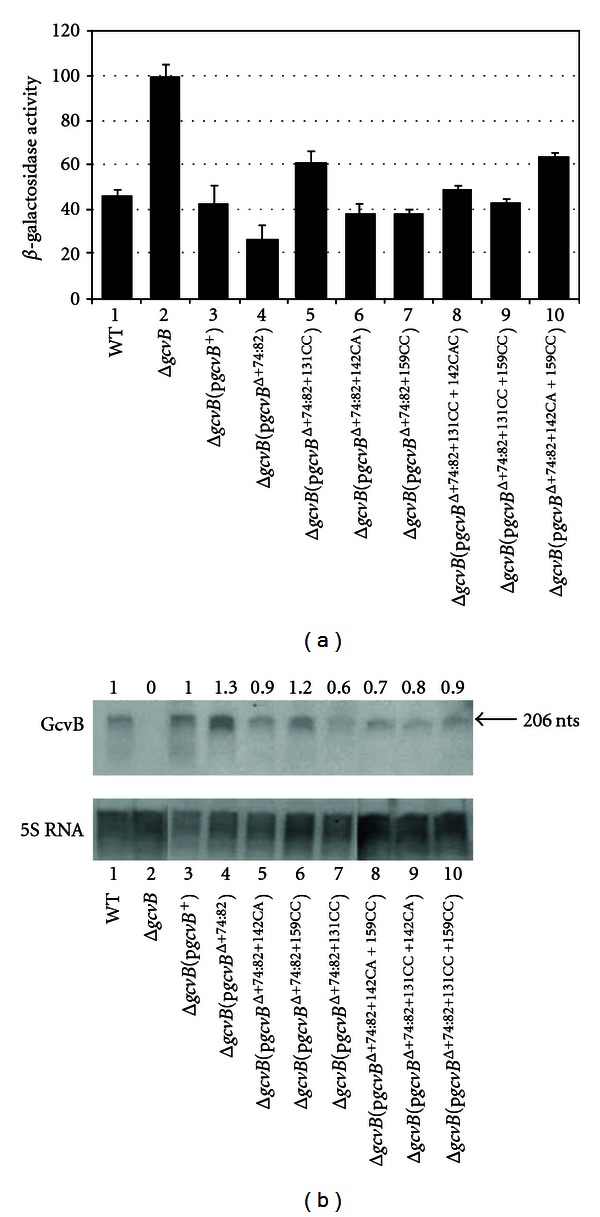
(a) Effects of the *gcvB* ^Δ+74 : 82^ allele plus additional mutations on *cycA*-*lacZ* expression. WT and Δ*gcvB* *λcycA*-*lacZ* lysogens transformed with the indicated plasmids were grown in LB (+Amp for transformants) to mid-log phase and assayed for *β*-galactosidase. (b) Northern analysis of GcvB. RNA was isolated from WT, Δ*gcvB,* or Δ*gcvB* transformed with the indicated plasmids and probed with either a DIG-labeled GcvB or 5S rRNA-specific DNA probe. Numbers above each lane indicate levels of GcvB relative to WT.

 To determine if the region distal to terminator t1 is able to repress *cycA*-*lacZ*, we constructed the *gcvB* ^Δ+74 : 82^ allele. This mutation removes the region of GcvB that precedes terminator t1 (Figure 1(c)) and shown above to play a role in regulation of *cycA*-*lacZ* in the presence of the *gcvB* ^t1↑^ allele (Figure 4(a)). Despite the size of the deletion, the mfold program [23, 24] predicts the remaining secondary structure of GcvB to remain intact. The *gcvB* ^Δ+74 : 82^ allele showed 1.8-fold better repression of *cycA*-*lacZ* than WT *gcvB* ^+^ (Figure 5(a), compare lanes 1 and 4). Next, we introduced the +131CC, +142CA and +159CC changes that do not alter *cycA*-*lacZ* expression in the full length GcvB [2], as well as combinations of these changes, into the *gcvB* ^Δ+74 : 82^ allele. The *gcvB* ^Δ+74 : 82+131CC^ and *gcvB* ^Δ+74 : 82+142CA+159CC^ mutations resulted in >2-fold higher levels of expression than the *gcvB* ^Δ+74 : 82^ mutation (Figure 5(a), compare lane 4 with 5 and 10). The remaining mutations showed smaller but statistically significant increases in expression compared to the *gcvB* ^Δ+74 : 82^ allele (Figure 5(a), compare lane 4 with lanes 6–9; *P* values of 0.036, 0.027, 0.004, and 0.014, resp.). A Northern analysis showed about the same levels of GcvB for each RNA sample tested (Figure 5(b)). The results suggest loss of repression or increased repression is not due to altered levels of the mutant RNAs.

 We also combined the *gcvB* ^Δ+74 : 82^ mutation with the *gcvB* ^t1↑^ allele. However, a Northern analysis of two separate RNA preparations from a strain carrying the p*gcvB* ^t1↑Δ+74 : 82^ allele showed only about 30% of the GcvB level found with p*gcvB* ^+^. The results suggest the *gcvB* ^t1↑Δ+74 : 82^ is unstable and was not pursued further.

### 3.5. Regulation Requires GcvB/*CycA* mRNA Interactions

**Figure 6:**
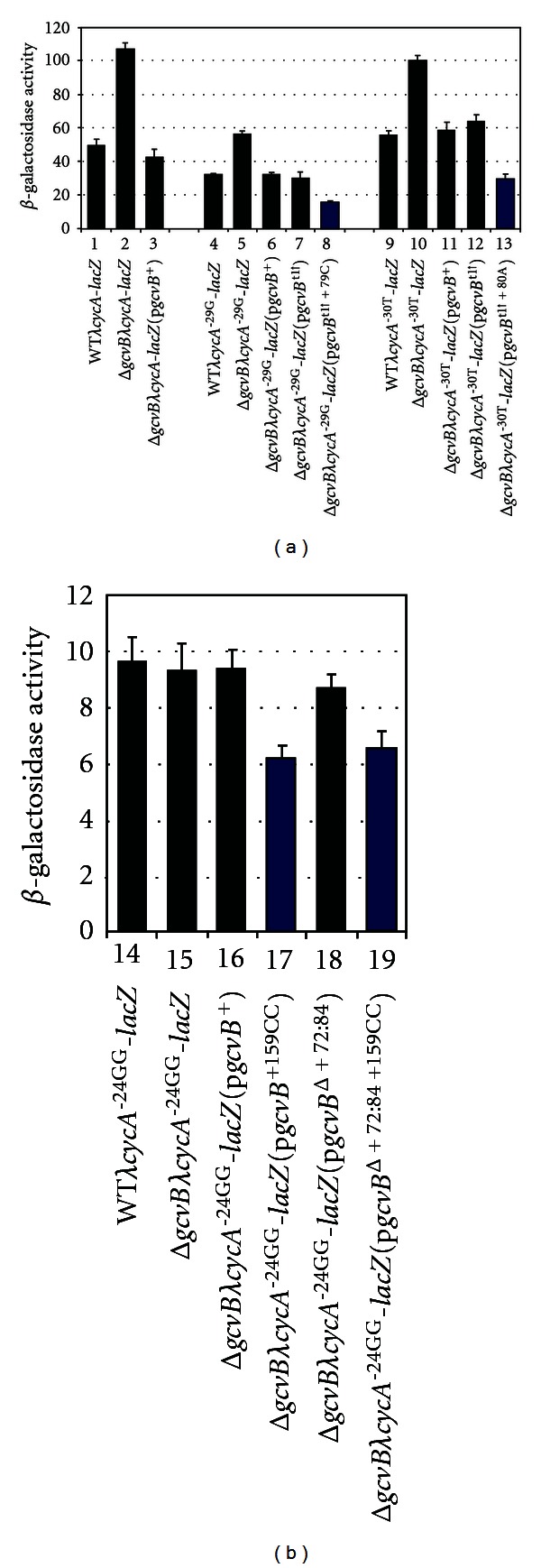
GcvB represses *cycA* mRNA by an antisense mechanism. WT and Δ*gcvB* *λcycA*-*lacZ*, *λcycA* ^−29G^-*lacZ*, *λcycA* ^−30T^-*lacZ,* and *λcycA* ^−24GG^-*lacZ* lysogens transformed with the indicated plasmids were grown in LB (+Amp for transformants) to mid-log phase and assayed for *β*-galactosidase.

 To confirm altered regulation is due to altered GcvB/*cycA* mRNA interactions, we constructed a *λcycA* ^−24GG^-*lacZ* fusion (an -AC- to -GG- change at nts −24, −25 relative to the *cycA* AUG start site); the changes reduce paring of *cycA* mRNA with both regions of GcvB complementary to *cycA* (Figures 1(b) and 1(c)). We also constructed *λcycA* ^−29G^-*lacZ* and *λcycA* ^−30T^-*lacZ* fusions (an -A- to -G- change at nt −29 and a -C- to -T- change at nt −30 relative to the *cycA* AUG start site, resp.); the changes reduce pairing of *cycA* mRNA with the +73 to +82 region of GcvB (Figures 1(b) and 1(c)). A WT*λcycA* ^−24GG^-*lacZ* lysogen had ~5.5-fold lower levels of expression than the WT**λ*cycA*-*lacZ* lysogen, suggesting the -GG- change affects translation efficiency (Figure 6, compare lines 1 and 14). The WT*λcycA* ^−24GG^-*lacZ* and Δ*gcvBλcycA* ^−24GG^-*lacZ* lysogens, as well as p*gcvB* ^+^ and p*gcvB* ^Δ+74 : 82^ complemented lysogens, showed essentially the same levels of expression, suggesting a complete loss of GcvB regulation (Figure 6, lines 14, 15, 16, and 18). However, the p*gcvB* ^+159CC^ and p*gcvB* ^+74 : 82+159CC^ alleles, that restore pairing with the *cycA* ^−24GG^ allele, repressed *cycA* ^−24GG^-*lacZ* expression about 1.5-fold (Figure 6, compare line 14 with lines 17 and 19). Although the changes are less than the normal 2-fold repression observed for *cycA* by GcvB, the results are statistically significant (*P* values of 0.0001 and 0.0028 relative to the WT*λcycA* ^−24GG^ lysogen, resp.) and suggest pairing of GcvB in the +124 to +161 region with *cycA* mRNA is required for repression.

 The WT *λcycA* ^−29G^-*lacZ* and *λcycA* ^−30T^-*lacZ* lysogens showed levels of expression similar to the WT**λ*cycA*-*lacZ* lysogen, suggesting the changes do not dramatically affect translation efficiency (Figure 6, compare lane 1 with lanes 4 and 9). In addition, *β*-galactosidase levels were about 2-fold higher in each Δ*gcvB* lysogen compared to its WT control and repression was restored in the Δ*gcvB*[p*gcvB* ^+^] and Δ*gcvB*[p*gcvB* ^t1↑^] transformants (Figure 6, compare lanes 4–7 and lanes 9–12). This is not unexpected since the *cycA* ^−29G^ and *cycA* ^−30T^ changes disrupt pairing with GcvB in the +73 to +82 region but do not disrupt pairing in the +124 to +161 region (Figures 1(b) and 1(c)). However, the p*gcvB* ^t1↑+79C^ and p*gcvB* ^t1↑+80A^ alleles, that restore pairing with the *cycA* ^−29G^ and *cycA* ^−30T^ alleles, respectively, increased repression an additional 2-fold (Figure 6, compare lanes 4 and 8 and lanes 9 and 13). The results suggest pairing of GcvB in the +73 to +82 region with *cycA* mRNA is also required for repression. The above results are in agreement with a model of genetic redundancy as a mechanism for *cycA* regulation by *E*. *coli* GcvB. 

## 4. Discussion

 In *E*. *coli* and *S*.* enterica*, GcvB has been shown to regulate multiple genes involved in amino acid and peptide transport [1–6]. However, most changes in GcvB predicted to disrupt pairing with target mRNAs had no significant effect on GcvB-mediated repression [2–4]. For the *cycA* mRNA, GcvB shows 2 regions of complementarity (Figures 1(b) and 1(c)). In this study, we tested if either region of complementarity is able to independently repress *cycA*-*lacZ*. The *gcvB* ^t1↑^ allele produces a truncated GcvB of ~134 nts and would remove most of the region from nt +124 to +161 complementary with *cycA* mRNA (Figure 1(a)). The *gcvB* ^t1↑^ allele showed better repression of *cycA*-*lacZ* than WT *gcvB* (Figure 4, lanes 3 and 4). The results suggest the region distal to terminator t1 is not necessary for repression of *cycA*-*lacZ* and possibly prevents full repression by GcvB. Mutations in *gcvB* in the +76 to +82 region that reduce complementarity with *cycA* mRNA (Figure 1(c)) result in a significant loss of repression in the presence of the *gcvB* ^t1↑^ mutation (Figure 4, compare lane 4 with lanes 5–8), and a change at nts +71 to +73 that increases complementarity results in increased repression (Figure 4, compare lanes 4 and 9). These results suggest the region of complementarity preceding terminator t1 is responsible for repression in the *gcvB* ^t1↑^ background. Of interest, these mutations do not alter GcvB repression in the full-length molecule [2]. The *gcvB* ^Δ+74 : 82^ allele, which removes the region of GcvB preceding terminator t1 involved in repression in the *gcvB* ^t1↑^ background (Figure 1(c)), also showed better repression of *cycA*-*lacZ* than WT *gcvB* (Figure 5(a), compare lanes 3 and 4). Thus, when the region of GcvB distal to terminator t1 is intact, the region of GcvB in the +74 to +82 region is not required for repression and appears to partially inhibit repression. Mutations in *gcvB* in the +131 to +160 region that do not alter GcvB repression in the full length GcvB [2] result in a significant loss of repression in the presence of the *gcvB* ^+74 : 82^ mutation (Figure 5(a), compare lane 4 with lanes 5–10). These results suggest the region of complementarity following terminator t1 is responsible for repression in the *gcvB* ^Δ+74 : 82^ background. Several of the mutations change the Con-II sequence (Figure 1(b)). However, other changes that result in a loss of repression fall outside of this region. Thus, although the Con-II sequence is likely involved in regulation of *cycA*, additional sequence is also required. In many bacteria, multiple largely redundant sRNAs control identical target mRNAs [27, 28]. In addition, a single sRNA can regulate many genes [29, 30]. Although most sRNAs use one region for basepairing, a few use independent regions to basepair with different target mRNAs. For example, two regions of DsrA are necessary for full activity on the *hns* and *rpoS* mRNAs [31, 32] and two different regions of FnrS basepair with different sets of target mRNAs [33]. The results in this study show that 2 regions of GcvB complementary with the same region of the *cycA* mRNA are able to independently basepair with the *cycA* mRNA and repress expression by an antisense mechanism. In addition, the results open the possibility that GcvB can bind simultaneously and repress two different mRNA molecules.

 Of interest, none of the mutations in the presence of the t1↑ allele or the Δ+74 : 82 allele resulted in a complete loss of GcvB repression of the *cycA*-*lacZ* fusion (Figures 4 and 5(a)). An examination of each mutant allele identified small regions that could still basepair with the *cycA* mRNA (not shown). If these small regions are involved in the repression observed, the results would suggest a high degree of flexibility in GcvB basepairing with target mRNAs. *S*. *enterica* GcvB also shows several redundant pairing regions with *cycA,* and *in vitro* experiments suggest several regions of GcvB independently inhibit translation initiation of *cycA* mRNA [6]. Thee results suggest genetic redundancy is a mechanism for regulation by GcvB.

 Since many of the genes that respond to GcvB are involved in transport of small peptides and amino acids, we hypothesize this is a survival mechanism to turn down transporters under conditions that favor the presence of toxic molecules that are also transported by these systems [2]. Another class of genes regulated by GcvB is involved in acid resistance (unpublished results) [34], suggesting GcvB plays a role in *E*. *coli* survival at low pH. Both of these environmental stresses would be encountered as *E*. *coli* moves from an external environment into the GI tract. We hypothesize the functions of the genes regulated by GcvB are crucial to cell survival when cells colonize the GI tract and the redundancy in GcvB prevents accidental loss of regulation of these genes by mutation or possible changes in GcvB structure induced by environmental conditions.

## Figures and Tables

**Table 1:** Strains, plasmids, and phage.

Strains*, plasmids, and phage	Relevant genotype	Source or reference
Strains		
GS162	WT	This lab
GS1144	Δ*gcvB *	[3]
GS1148	Δ*hfq *	[13]
Plasmids		
pGS341	Single-copy vector + WT *gcvA *	[17]
pGS594	Single-copy vector + WT *gcvB *	This lab
pGS596	pGS594 with a -TGT- to -CCC- change of bps +71 to +73 in *gcvB *(p*gcvB* ^+71CCC^)**	[3]
pGS602	pGS594 with a -TGT- to -AAA- change of bps +76 to +78 in *gcvB* (p*gcvB* ^+76AAA^)	[3]
pGS629	pGS594 with a -TGTT- to -CCCA- change of bps +79 to +82 in *gcvB* (p*gcvB* ^+79CCCA^)	[4]
pGS634	pGS594 with a -TG- to -CA- change of bps +142 and +143 and a -TG- to -CC- change of bps +159 and +160 in *gcvB* (p*gcvB* ^+142CA+159CC^)	[2]
pGS642	Single-copy vector + *gcvB* ^t1↑^ allele (see Figure 2 for bp changes) (p*gcvB* ^t1↑^)	This study
pGS644	pGS634 with a -T- to -C- change of bp +79 in *gcvB* (p*gcvB* ^+142CA+159CC+79C^)	This study
pGS645	pGS634 with a -G- to -A- change of bp +80 in *gcvB* (p*gcvB* ^+142CA+159CC+80A^)	This study
pGS647	pGS642 with -TGT- to -CCC- change of bps +71 to +73 in *gcvB *(p*gcvB* ^t1↑+71CCC^)	This study
pGS649	pGS642 with a -TGT- to -AAA- change of bps +76 to +78 in *gcvB *(p*gcvB* ^t1↑+76AAA^)	This study
pGS653	pGS642 with a -TGTT- to -CCCA- change of bps +79 to +82 in *gcvB* (p*gcvB* ^t1↑+79CCCA^)	This study
pGS655	pGS642 with a -T- to -C- change of bp +79 in *gcvB* (*gcvB* ^t1↑+79C^)	This study
pGS656	pGS642 with a -G- to -A- change of bp +80 in *gcvB* (p*gcvB* ^t1↑+80A^)	This study
pGS680	pGS594 with a deletion from bp +74 to +82 in *gcvB* (p*gcvB* ^Δ+74 : 82^)	This study
pGS682	pGS680 with a -TG- to -CA- change of bps +142 and +143 in *gcvB* (p*gcvB* ^Δ+74 : 82+142CA^)	This study
pGS683	pGS680 with a -TG- to -CC- change of bps +159 and +160 in *gcvB *(p*gcvB* ^Δ+74 : 82+159CC^)	This study
pGS684	pGS680 with a -TT- to -CC- change of bps +131 and +132 in *gcvB *(p*gcvB* ^Δ+74 : 82+131CC^)	This study
pGS688	pGS680 with the *gcvB* ^t1↑^ change (p*gcvB* ^t1↑Δ+74 : 82^)	This study
pGS697	pGS680 with a -TG- to -CA- change of bps +142 and +143 and a -TG- to -CC- change of bps +159 and +160 in *gcvB* (p*gcvB* ^Δ+74 : 82+142CA+159CC^)	This study
pGS698	pGS680 with a -TT- to -CC- change of bps +131 and +132 and a -TG- to -CA- change of bps +142 and +143 in *gcvB* (p*gcvB* ^Δ+74 : 82+131CC+142CA^)	This study
pGS699	pGS680 with a -TT- to -CC- change of bps +131 and +132 and a -TG- to CC change of bps +159 and +160 in *gcvB* (p*gcvB* ^Δ+74 : 82+131CC+159CC^)	This study
Phage		
*λ*gt2	*λ* cloning vector; *c*I857 repressor	[18]
*λcycA*-*lacZ *	*λ* vector carrying WT *cycA*-*lacZ* translational fusion	[2]
*λcycA* ^−24GG^-*lacZ *	*λ* vector carrying a *cycA* ^−24GG^-*lacZ* translational fusion with an -AC- to -GG- change at nts −24 and −25	This study
*λcycA* ^−29G^-*lacZ *	*λ* vector carrying a *cycA* ^−29G^-*lacZ* translational fusion with an -A- to -G- change at nt −29	This study
*λcycA* ^−30T^-*lacZ *	*λ* vector carrying a *cycA* ^−39T^-*lacZ* translational fusion with a -C- to -T- change at nt −30	This study

*All strains also carry the *pheA905 thi araD129 rpsL150 relA1 deoC1 flbB5301 ptsF25 rbsR* mutations.

**Numbering for *gcvB* mutations is based on the transcription initiation site as +1. Numbering for the *cycA* fusions and mutations is based on the A residue in the AUG translation initiation codon as +1 with bases upstream assigned negative values.
